# Prophylaxis

**Published:** 1901-10

**Authors:** J. H. Benton

**Affiliations:** New Bern, N. C.


### PROPHYLAXIS.[1]

BY DR. J. H. BENTON, NEW BERN, N. C.

By prophylaxis is meant preservative or preventive treatment. Within every individual is fixed an incentive to strive to maintain his earthly existence, even when surrounding circumstances make it apparent that it would be better to let life pass away. The prolongation of life has ever been and ever will be a question for rumination. Naturally, the young might seem to be the only legitimate claimants for prolonged life, but we see the old grasping just as ardently for existence; and as the years go passing rapidly by, the aged person will strive to restrain the wasting of the vital forces and employ every so-called specific that may offer hope of longer earthly existence. To-day, as it ever will be, we see in earnest solicitation everlasting life, if not endless youth. The inclination to cling to life is not affected by the changes of time, and the followers of Brown-Séquard using for the perpetuation of human life and powers the elixir of life and those using the phospho-albumin treatment are as enthusiastic as are those hoping to reach the fount of endless youth.

Recently it has been stated that some great electrician has promised an electric treatment which shall practically banish death till the time when it may be sought voluntarily.

Early in the nineteenth century medical science turned away from the study of theory to the study of life,—anatomy, physiology, pathology, and sanitation,—and in later years is giving attention to the study of physiological and pathological chemistry. "The

---

[1] Read before the North Carolina Dental Society, Morehead City, N. C., June 26, 1901.

proper study of mankind is man." With the discovery of bacteria there has been accomplished such miracles in medicine and surgery as had never been dreamed of by mortal man. From all progressive countries have been successfully excluded cholera, yellow fever, and the leprosy plague; and typhus fever, which was formerly the scourge of every great city, is a disease now rarely named in the catalogue of causes producing death; and even typhoid fever for the past decade or two has been rapidly retreating before the advances of science. The mortality of diphtheria has diminished wonderfully since the introduction of the antitoxic treatment in 1892. In well-governed countries small-pox is almost unknown except in the slums of seaport cities: a rare disease now and scarcely ever epidemic—a grand triumph for preventive medicine. Consumption, by far the worst of all human plagues, is now yielding to the skill of the practitioner and sanitation, and it is hoped will eventually be driven out of existence.

It is now a well-established fact that, outside of imperfection of development and structure, micro-organisms are the cause of decay of the teeth.

Another fact equally well established is that the destructive action of bacteria can be arrested and the bacteria themselves destroyed by the use of suitable antiseptics and germicides.

The broad statement has been advanced that if the mouth could at all times be kept antiseptically clean no teeth would decay. The truth of this no person can gainsay, but, unfortunately, such a condition cannot be maintained. If the dental profession expects or hopes to occupy a prominent position among the professions, workers along this line must spring from our ranks, men whose lives and unceasing efforts shall be directed to the discovery of a system of dental prophylaxis. Within a quarter of a century marvellous achievements have been made by bacteriologists, whose unceasing efforts have been constantly encouraged by progress and success. The number of this noble band of workers should be annually increased from the young members of the dental profession, and those who have no talent for such work should give their aid, encouragement, and approval.

The prevention of disease may be attempted, it is very evident, in two different ways. We may attempt to avoid or remove the cause of disease, or we may try to make the body less susceptible to the action of such agents as produce disease. The success which has been attained in modifying plants and lower animals by regimen and breeding makes it more than probable that man can in a similar manner be improved; physically, he is nothing more than a specialized animal, and his relation with the forces of the universe are not altogether unlike those of less specialized animals.

Among the many conditions requisite to retain and enjoy the blessings of health and immunity from disease that may be mentioned as of importance from a hygienic point of view are the local habitation or home of the individual, drainage of ground, thorough ventilation of the premises, clothing suitable to the season and climate and the degree of exposure of the person, sufficient bodily out-door exercise, taken daily in sunshine if possible, food of proper quality and quantity. "And God said, Behold, I have given you every herb bearing seed,"—that is, all the cereal plants, such as corn, wheat, rye, etc., whose peculiar distinction and characteristic it is to produce seed,—"and every tree in the which is the fruit of a tree yielding seed; to you it shall be meat." In these words God assigns and points out to man the food most suitable for him. It is plainly intended that he should subsist on vegetable food—herbs, grains, and fruits. These only were allowed to and used by man in his first estate. The abstinence from animal food exists in the traditions of all nations as one of the characteristics of their golden age, or the age of innocence. All food should be properly prepared and properly masticated, and taken with regularity, followed by rest, repose, and sleep. The process of nutrition and restoration of the nervous and muscular system takes place mainly through sleep. Cleanliness is most essential to hygiene—cleanliness of the person, especially of the mouth; cleanliness in all things pertaining to ourselves, our work, and our surroundings. A clean conscience will exercise a wonderful influence for good.

